# 6-Gingerol Protects against Nutritional Steatohepatitis by Regulating Key Genes Related to Inflammation and Lipid Metabolism

**DOI:** 10.3390/nu7020999

**Published:** 2015-02-04

**Authors:** Thing-Fong Tzeng, Shorong-Shii Liou, Chia Ju Chang, I-Min Liu

**Affiliations:** Department of Pharmacy & Graduate Institute of Pharmaceutical Technology, Tajen University, Yanpu Township, 90741 Pingtung County, Taiwan; E-Mails: d850084@yahoo.com.tw (T.-F.T.); ssliou@tajen.edu.tw (S.-S.L.); 7419417@yahoo.com.tw (C.J.C.)

**Keywords:** 6-gingerol, inflammatory cytokine, methionine and choline-deficient (MCD) diet, nuclear transcription factor κB (NF-κB), steatohepatitis

## Abstract

Non-alcoholic fatty liver disease, including non-alcoholic steatohepatitis (NASH), appears to be increasingly common worldwide. The aim of the study was to investigate the effects of 6-gingerol ((*S*)-5-hydroxy-1-(4-hydroxy-3-methoxyphenyl)-3-decanone), a bioactive ingredient of plants belonging to the *Zingiberaceae* family, on experimental models of NASH. In HepG2 cells, 6-gingerol (100 μmol/L) treatment inhibited free fatty acids mixture (0.33 mmol/L palmitate and 0.66 mmol/L oleate)-induced triglyceride and inflammatory marker accumulations. Male C57BL/6 mice were fed with a methionine and choline-deficient (MCD) diet to induce steatohepatitis. After four weeks of MCD diet feeding, the mice were dosed orally with 6-gingerol (25, 50 or 100 mg/kg/day) once daily for another four weeks. 6-Gingerol (100 mg/kg/day) attenuated liver steatosis and necro-inflammation in MCD diet-fed mice. The expressions of inflammatory cytokine genes, including those for monocyte chemoattractant protein-1, tumor necrosis factor-α, and interleukin-6, and nuclear transcription factor (NF-κB), which were increased in the livers of MCD diet-fed mice, were attenuated by 6-gingerol. 6-Gingerol possesses a repressive property on hepatic steatosis, which is associated with induction of peroxisome proliferator-activated receptor α. Our study demonstrated the protective role of 6-gingerol in ameliorating nutritional steatohepatitis. The effect was mediated through regulating key genes related to lipid metabolism and inflammation.

## 1. Introduction

Non-alcoholic fatty liver disease (NAFLD) is one of the most prevalent forms of chronic liver disease in developed countries, and is frequently associated with obesity, metabolic syndrome, and type 2 diabetes. The spectrum of NAFLD includes steatosis to non-alcoholic steatohepatitis (NASH), which can lead to cirrhosis and hepatocellular cancer [1]. The development of NASH is frequently described by a “two-hit” mechanism, in which liver steatosis constitutes the “first hit” and is accompanied by obesity as well as metabolic disruptions that cause excessive hepatic lipid accumulation. Liver steatosis then increases the susceptibility of the liver to a “second hit” in the form of oxidative stress or pro-inflammatory insults that result in NASH [2]. Therapeutic approaches that can suppress oxidative stress and/or pro-inflammatory responses should thus prevent against the development of NASH.

Numerous drugs have been tested for their ability to ameliorate fatty liver and NASH. These treatments have diverse pharmacological activities, such as improving insulin sensitivity, stimulating lipid oxidation, and reducing *de novo* lipogenesis [3]. However, therapies for these conditions that could limit hepatic injury and the associated inflammation would be particularly appealing. Of the various phytochemicals showing various biochemical and pharmacologic activities, 6-gingerol ((*S*)-5-hydroxy-1-(4-hydroxy-3-methoxyphenyl)-3-decanone), a major pharmacologically active component of ginger, has been reported to exhibit antioxidant, anti-inflammatory, anticancer, analgesic and antiplatelet effects, among others [4,5,6]. It has been demonstrated that 6-gingerol has significant potential as an anti-hyperglycemic, lipid lowering, and antioxidant agent for the treatment of type 2 diabetes [7]. Additionally, it has an inhibitory effect on xanthine oxidase, which is responsible for the generation of reactive oxygen species like superoxide anion [8]. 6-Gingerol can also work against cytokine-induced inflammation and oxidative stress in hepatocellular carcinoma HuH7 cells [9]. Although 6-gingerol might be effective in the treatment of NASH, this issue has not yet been explored in the literature.

It is well known that free fatty acids (FFAs) induce hepatic lipid accumulation in NASH [2]. FFAs-induced lipid accumulation in hepatocytes is a commonly used model to study hepatic steatosis [10]. Although the amount of lipid accumulation caused by oleic acid is less than that due to palmitic acid, oleic acid does not exhibit cytotoxicity [11]. To achieve maximal fat over-accumulation without cytotoxicity, a mixture of FFAs consisting of a low proportion of palmitic acid (palmitic acid:oleic acid = 1:2 ratio) was used to induce hepatic lipid in human hepatoma HepG2 cells [11]. The effects of 6-gingerol against NASH were thus characterized in human hepatoma HepG2 cells that had been incubated with a mixture of palmitate and oleate [11].

The animal models that have been used to investigate drugs directed against NASH were based on genetic defects (ob/ob mice or Fa/Fa rats) or feeding a methionine and choline-deficient (MCD) diet to rodents [12]. The MCD diet model has been verified as a more suitable experimental model to study this human disorder, especially with regard to the histopathological features of severe pericentral steatosis and necro-inflammation [13]. Therefore, we also conducted this study to determine if 6-gingerol could prevent against the development of steatosis and limit the expression of inflammatory genes in an MCD diet-induced hepatosteatosis animal model.

## 2. Materials and Methods

### 2.1. Materials

6-Gingerol (≥98%), ciprofibrate, sodium palmitate, sodium oleate and Oil Red O were obtained from Sigma-Aldrich, Inc. (St. Louis, MO, USA). 3-(4,5-Dimethylthiazol-2-yl)-5-(3carboxymethoxyphenyl)-2-(4-sulfophenyl)-2H-tetrazolium (MTS) and DNase were obtained from Promega (Madison, WI, USA). The Bio-Rad protein assay kit was purchased from Bio-Rad Laboratories Inc. (Hercules, CA, USA). The enzyme-linked immunosorbent assay (ELISA) kits for the determination of monocyte chemoattractant protein-1 (MCP-1), tumor necrosis factor-α (TNF-α), and interleukin (IL)-6 were obtained from Abcam Inc. (Cambridge, MA, USA). A triglyceride (TG) colorimetric assay kit and kits for determining plasma levels of glucose and total cholesterol (TC) were obtained from Cayman Chemical Company (Ann Arbor, MI, USA). Kits for determining plasma concentrations of alanine aminotransferase (ALT; EC 2.6.1.2) and aspartate aminotransferase (AST; EC 2.6.1.1) were purchased from Teco Diagnostics (Anaheim CA, USA). The nuclear extract kit and TransAM^®^ NF-κB p65 transcription factor assay kit were obtained from Active Motif (Tokyo, Japan). Inhibitory kappa B (IκBα) antibody, NF-κB p65 antibody, α-tubulin antibody and horseradish peroxidase-conjugated secondary antibodies were purchased from Santa Cruz Biotechnology, Inc. (Santa Cruz, CA, USA). The enhanced chemiluminescence (ECL) detection system was obtained from Amersham Biosciences (Buckinghamshire, UK). The Trizol reagent, RNaseOUT recombinant ribonuclease inhibitor and SuperScript III reverse transcriptase were purchased from Invitrogen (Boston, MA, USA).

### 2.2. Cell Culture and Treatments

Human hepatoma HepG2 cells were obtained from the Bioresource Collection and Research Center (BCRC 60025) of the Food Industry Research and Development Institute (Hsinchu, Taiwan). These cells were cultured in minimum essential medium that contained fetal bovine serum (10% by volume), l-glutamate (2 mmol/L), sodium pyruvate (1 mmol/L), penicillin (100 U/mL), streptomycin (100 μg/mL), and sodium pyruvate (1 mmol/L) at 37 °C in a humidified atmosphere with 5% CO_2_. Cells were grown to 70% confluence and incubated in serum-free medium overnight prior to treatment. After starvation for 24 h, HepG2 cells were exposed to a FFAs mixture (0.33 mmol/L palmitate and 0.66 mmol/L oleate) for 24 h, and then were incubated for another 24 h with vehicle, or with 6-gingerol at concentrations of 25, 50 μmol/L, or 100 μmol/L, or with 100 μmol/L of peroxisome proliferator-activated receptor-α (PPARα) agonist ciprofibrate [9,14]. The vehicle (distilled water) used to prepare the test medication solutions was given at the same volume. The cells were also exposed to FFAs during incubation with the test compounds. Cells supplemented with medium without any treatment served as the untreated control. The concentrations used were selected based on a previous report, which showed that 6-gingerol protected hepatocellular carcinoma HuH7 cells against IL-1β-induced inflammatory insults [9].

Cell viability was assayed using MTS. Treating HepG2 cells with a FFAs mixture did not inhibit their growth (cell viability was 99.9%). 6-Gingerol (100 μmol/L) and ciprofibrate (100 μmol/L) were not cytotoxic for HepG2 cells, and the cell viabilities were 99.8% and 99.7%, respectively. The FFAs mixture-induced lipid accumulation in HepG2 cells was evaluated by Oil Red O staining and by measuring the cellular TG contents.

### 2.3. Oil Red O Staining

HepG2 cells were rinsed with cold phosphate buffered saline and fixed for 30 min in 10% paraformaldehyde. After washing with 60% isopropanol, cells were stained for at least 1 h in a freshly diluted Oil Red O solution (6 parts Oil Red O stock solution and four parts H_2_O; Oil Red O stock solution was 0.5% Oil Red O in isopropanol). Excess stain was removed, cells were washed with 60% isopropanol, and each group of cells was photographed. Stained lipid droplets were then extracted with isopropanol for quantification by measuring absorbance at 490 nm [15].

### 2.4. Measurements of TG in HepG2 Cells

HepG2 cells were lysed with 1% Triton X-100 in PBS, after which cellular TG levels were determined using enzymatic colorimetric assay kits. Cell protein concentrations were determined using a Bio-Rad protein assay kit, using bovine serum albumin as the standard. Cellular TG levels were normalized to cellular protein contents.

### 2.5. Measurements of Cytokines in HepG2 Cells

After treatments, cells were centrifuged at 10, 000× *g* at 4 °C for 10 min, and the supernatants were stored at −20 °C until analyzed. Secretory levels of inflammatory cytokines in cell-free culture supernatants, including MCP-1, TNF-α, and IL-6, were determined using commercial ELISA kits. The colors that were produced were determined by measuring optical density (OD) at 450 nm with a microtiter plate reader (Molecular Devices Corp., Sunnyvale, CA, USA). A standard curve was prepared on each assay plate using serially diluted recombinant proteins.

### 2.6. Animal and Experimental Protocols

C57BL/6 mice (six-week-old males) were obtained from the National Laboratory Animal Center (Taipei, Taiwan). They were maintained in a temperature-controlled room (25 ± 1 °C) on a 12 h:12 h light-dark cycle (lights on at 6:00 AM) in our animal center. Food and water were provided *ad libitum*. MCD and methionine- and choline-sufficient (MCS) diets were purchased from Dyets, Inc. (#518810 and #518754, respectively; Bethlehem, PA, USA). Both diets contained similar amounts of nutrients (14.2% protein, 15% fat, 3.09% ash, and 5% fiber), except that methionine and choline were not included in the MCD diet, whereas 1.70 g/kg of methionine and 14.48 g/kg of choline bitartrate were provided in the MCS diet. All animal procedures were conducted according to the Guide for the Care and Use of Laboratory Animals of the National Institutes of Health, as well as guidelines of the Animal Welfare Act. These studies were approved by the Institutional Animal Care and Use Committee (IACUC) of Tajen University (approval number: IACUC 102-16; approval date: 24 December 2013).

After consuming a MCD diet for four weeks, mice were administered 6-gingerol once daily via oral gavage at doses of 25, 50, or 100 mg/kg in 1.5 mL/kg of distilled water. These dosages were selected based on a previous report that demonstrated that 6-gingerol was potentially effective for reducing hyperlipidemia in diabetic db/db mice [7]. Another group of MCD diet-fed mice was orally administered ciprofibrate (10 mg/kg/day) dissolved in distilled water, a dose based on a study that indicated that long-term treatment ameliorated hyperlipidemia in hypertriglyceridemic mice [14]. Vehicle-treated mice received 1.5 mL/kg of distilled water only.

After the additional four-week treatment, animals were weighed, fasted for 12 h, and then anesthetized with ketamine. Blood samples from the inferior vena cava were collected for analysis, after which the liver was removed, rinsed with physiological saline, and immediately stored at −80 °C in liquid nitrogen until assayed. Relative liver weight was the liver weight divided by body weight. Other hepatic tissues were fixed in 10% neutralized formalin for histology.

### 2.7. Biochemical Analysis

The systemic biochemical analyses included glycemia, TG, and TC. Liver integrity was assessed by measuring blood levels of enzymes AST, and ALT.

### 2.8. Measurements of Hepatic Lipids and Cytokines

Hepatic lipid contents were determined using fresh liver samples. A liver specimen (1.25 g) was homogenized with chloroform/methanol (1:2, 3.75 mL) and well-mixed with chloroform (1.25 mL) and distilled water (1.25 mL). After centrifuging at 1500× *g* for 10 min, the lower, clear organic phase was transferred to a new glass tube and lyophilized. This lyophilized powder was dissolved in chloroform/methanol (1:2) and stored at −20 °C for less than three days [16]. Hepatic TC and TG levels in the lipid extracts were assayed using the same diagnostic kits used for plasma analysis.

For hepatic cytokine measurements, liver samples were homogenized in 10 mmol/L of Tris-HCl buffered solution (pH 7.4) that contained NaCl (2 mol/L), EDTA (1 mmol/L), Tween 80 (0.01%), and PMSF (1 mmol/L), and then centrifuged at 9000× *g* at 4 °C for 30 min. The supernatants were used for cytokine determination using the same diagnostic kits used to measure cytokines in HepG2 cells.

### 2.9. Hepatic Histological Analysis

At the time of sacrifice, a liver was perfused with phosphate buffered saline via the portal vein. After removing the liver, a section approximately 4 mm^2^ in size was fixed in PAF and embedded in paraffin. Paraffin-embedded sections (5 μm) were stained with hematoxylin and eosin to evaluate the degree of hepatic steatosis. Liver tissues were scored for hepatic steatosis: 0 (no steatosis), 1 (1%–25%), 2 (26%–50%), 3 (51%–75%), and 4 (76%–100%) of affected hepatocytes. These tissues were also scored for necro-inflammation: 0 (no inflammation), 1 (mild lobular/portal inflammation), 2 (moderate lobular/portal inflammation), and 3 (severe lobular/portal inflammation) [17]. All slides were scanned at a magnification of 200× using Image Pro Plus 7.0 software (Media Cybernetics, Inc., Rockville, MD, USA) under a light microscope (Olympus BX51 microscope; Tokyo, Japan).

### 2.10. NF-κB Activity

Nuclear extracts of livers from the experimental animals were prepared using a nuclear extract kit. NF-κB activity was determined in the nuclear extracts (20 μg) using a NF-κB p65 transcription factor assay kit, following the manufacturer’s instructions.

### 2.11. Western Blotting

Liver tissues were homogenized in 1 mL of ice-cold hypotonic buffer A (10 mmol/L of HEPES, 10 mmol/L of KCl, 2 mmol/L of MgCl_2_, 1 mmol/L of DTT, 0.1 mmol/L of EDTA, and 0.1 mmol/L of phenylmethylsulfonylfluoride; pH 7.8). Cells were then lysed with 12.5 μL of 10% Nonidet P-40. The homogenates were centrifuged, and supernatants that contained the cytoplasmic extracts were stored frozen at −80 °C. A nuclear pellet was resuspended in 25 μL of ice-cold nuclear extraction buffer. After 30 min with intermittent mixing, the extract was centrifuged, and supernatants that contained nuclear extracts were secured.

Prior to immunoblotting, the protein concentration of each tissue sample was determined using a Bio-Rad protein assay kit, using bovine serum albumin as the standard to ensure equal loading among lanes. Cytosolic (70 μg total protein) and nuclear (50 μg total protein) extracts were separated on a 7.5%–15% polyacrylamide gel and then transferred electrophoretically to nitrocellulose membranes. Membranes were blocked with 5% non-fat dry milk in Tris-buffered saline Tween (20 mmol/L of Tris, pH 7.6, 137 mmol/L of NaCl, and 0.1% Tween 20) at room temperature for 3 h and then incubated at 4 °C overnight with IκBα and NF-κB p65 primary antibodies. The level of α-tubulin was estimated for equal sample loading. Membranes were washed three times with Tris-buffered saline Tween 20 (TBST) and incubated at room temperature for 1 h with appropriate horseradish peroxidase-conjugated secondary antibodies. After three additional TBST washes, immunoreactive bands were visualized using an ECL method according to the manufacturer’s instructions. Band densities were determined using ATTO Densitograph Software (ATTO Corporation, Tokyo, Japan). All experimental sample values were expressed relative to this adjusted mean value. Tissue sections were sampled for four independent experiments.

### 2.12. Hepatic mRNA Expression

To examine gene expression, total RNA was extracted from frozen liver samples (100 mg) using Trizol reagent. RNA was quantified by absorbance at A260, and its integrity was verified by agarose gel electrophoresis using ethidium bromide for visualization. For a reverse transcriptase reaction, 1 μg of total RNA per sample and 8.5 μg/μL of random hexamer primers were heated to 65 °C for 5 min, and then quenched on ice. This mixture was combined with 500 μmol/L each of dATP, dTTP, dCTP, and dGTP, 10 mmol/L of DTT, 20 mmol/L of Tris-HCl (pH 8.4), 50 mmol/L of KCl, 5 mmol/L of MgCl_2_, 40 units of RNaseOUT recombinant ribonuclease inhibitor, and 100 units of SuperScript III reverse transcriptase. Samples were treated with DNase at 37 °C for 20 min in a GeneAmp 9700 Thermal Cycler (Applied Biosystems; Foster City, CA, USA) and then held at 4 °C. After aliquots were taken for immediate use in PCR, the remaining cDNA was stored at −20 °C.

mRNA expression was determined by quantitative real-time reverse transcription polymerase chain reaction (RT-PCR) using a fluorescent temperature Lightcycler 480 (Roche Diagnostics; Mannheim, Germany). The following primer sequences were used: 5′-TCTCTTCCTCCACCACTATGCA-3′ (forward) and 5′-GGCTGAGACAGCACGTGGAT-3′ (reverse) for MCP-1; 5′-ATGGATCTCAAAGACAACCA-3′ (forward) and 5′-TCCTGGTATGAAATGGCAAA-3′ (reverse) for TNF-α; 5′-AAAAGTCCTGATCCAGTTC-3′ (forward) and 5′-GAGATGAGTTGTCATGTCC-3′ (reverse) for IL-6; 5′-CCTTAACCCTGAGATCCCGTAGA-3′ (forward) for acyl-CoA:diacylglycerol acyltransferase 2 (DGAT2); 5′-CGTCCTGGCCTTCTAAACGTAG-3′ (forward and 5′- AGCCCATAAAAGATTTC1GCAAA-3′ (reverse)) and 5′-CCTGTAGATCTCCTGCAGTAGCG-3′ (reverse) for peroxisome proliferator-activated receptor α (PPARα); 5′-TGTGATGGTGGGAATGGGTCAG-3′ (forward) and 5′-TTTGATGTCACGCACGAT TTCC-3′ (reverse) for β-actin. Primers were designed using Primer Express Software version 2.0 System (Applied Biosystems; Foster City, CA, USA). A PCR reaction was run using the following cycling protocol: 95 °C for 5 min, and 45 cycles of 95 °C for 5 s, 58 °C for 15 s, and 72 °C for 20 s. Dissociation curves were run after amplification to identify specific PCR products. mRNA expression levels were normalized to β-actin mRNA levels and calculated using the delta-delta Ct method [18].

### 2.13. Statistical Analysis

Results are expressed as means ± standard deviations (SD). Statistical comparisons were made using one-way analysis of variance (ANOVA). Dunnett range *post-hoc* comparisons were used to determine the source of significant differences, as appropriate. A non-parametric Kruskal-Wallis test was used for the histological studies, and Mann-Whitney’s *U* test was used to compare results within groups. SigmaPlot (Version 11.0) was used for the statistical analysis. *p*-Values of <0.05 were considered significant.

## 3. Results

### 3.1. Effects on Intracellular Lipid Accumulation and Inflammatory Cytokines in FFA Mixture-Treated HepG2 Cells

**Figure 1:**
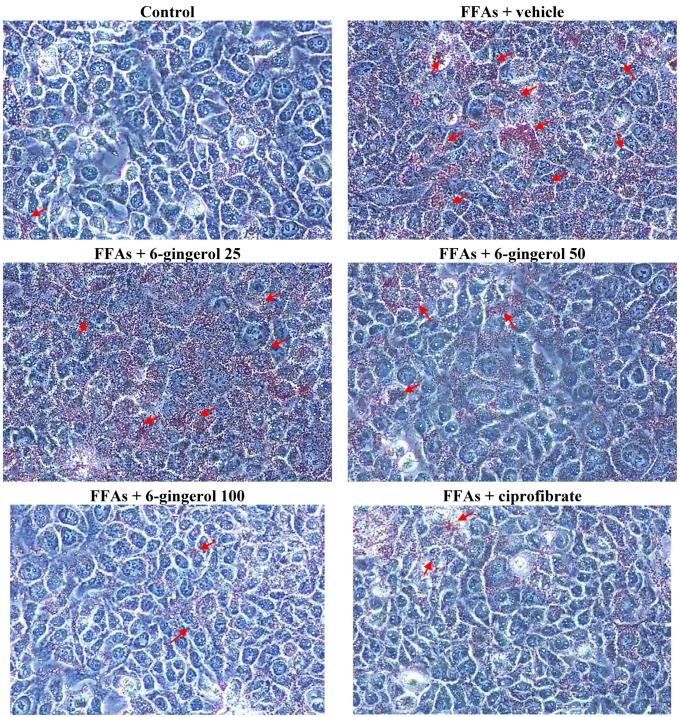
Representative images of FFAs mixture-treated HepG2 cells stained with Oil Red O after different treatments. HepG2 cells were exposed to a FFAs mixture (0.33 mmol/L palmitate and 0.66 mmol/L oleate) for 24 h, and were then incubated for another 24 h with 6-gingerol at concentrations of 25 μmol/L (6-gingerol 25), 50 μmol/L (6-gingerol 50), or 100 μmol/L (6-gingerol 100), or with 100 μmol/L of ciprofibrate (ciprofibrate). The vehicle (distilled water) used to prepare the test medication solutions was given at the same volume. Cells supplemented with medium without any treatment served as control. Cells were observed by light microscopy at a magnification of 400×. Arrows indicate fat droplets.

**Figure 2:**
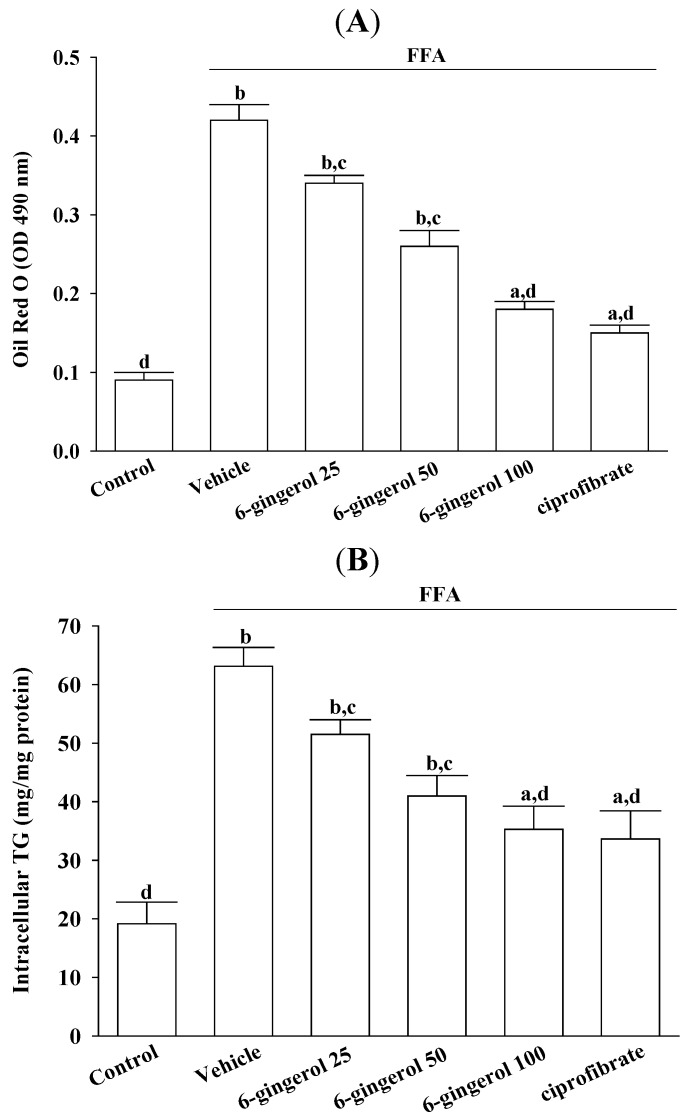
Effects of different treatments on FFAs mixture-induced cellular lipid accumulation in HepG2 cells. HepG2 cells were exposed to a FFAs mixture (0.33 mmol/L palmitate and 0.66 mmol/L oleate) for 24 h, and were then incubated for another 24 h with 6-gingerol at concentrations of 25 μmol/L (6-gingerol 25), 50 μmol/L (6-gingerol 50), or 100 μmol/L (6-gingerol 100), or with 100 μmol/L of ciprofibrate (ciprofibrate). The vehicle (distilled water) used to prepare the test medication solutions was given at the same volume. Cells supplemented with medium without any treatment served as the control. (**A**) Stained lipid droplets were analyzed by spectrophotometry; (**B**) Total intracellular TG contents determined by an enzymatic colorimetric method. ^a^ *p* < 0.05 and ^b^ *p* < 0.01 compared to untreated control results. ^c^ *p* < 0.05 and ^d^ *p* < 0.01 compared to results for FFAs mixture-treated cells.

HepG2 cells that were treated for 24 h with a FFAs mixture exhibited significant lipid droplet accumulation as compared with untreated cells (Figure 1 and Figure 2). Pre-incubating these cells with 6-gingerol significantly reduced FFAs mixture-induced lipid deposition, and the most effective inhibition of lipid accumulation was found at a 6-gingerol concentration of 100 μmol/L (Figure 1 and Figure 2). Treatment with an FFAs mixture resulted in a significant increase in TG contents as compared with untreated cells, and this was significantly attenuated by pre-treatment with 6-gingerol (100 μmol/L; Figure 2). The lipid deposition and increases in TG contents in FFAs mixture-treated HepG2 cells were also abolished by pre-incubation with ciprofibrate (100 μmol/L; Figure 2).

**Figure 3:**
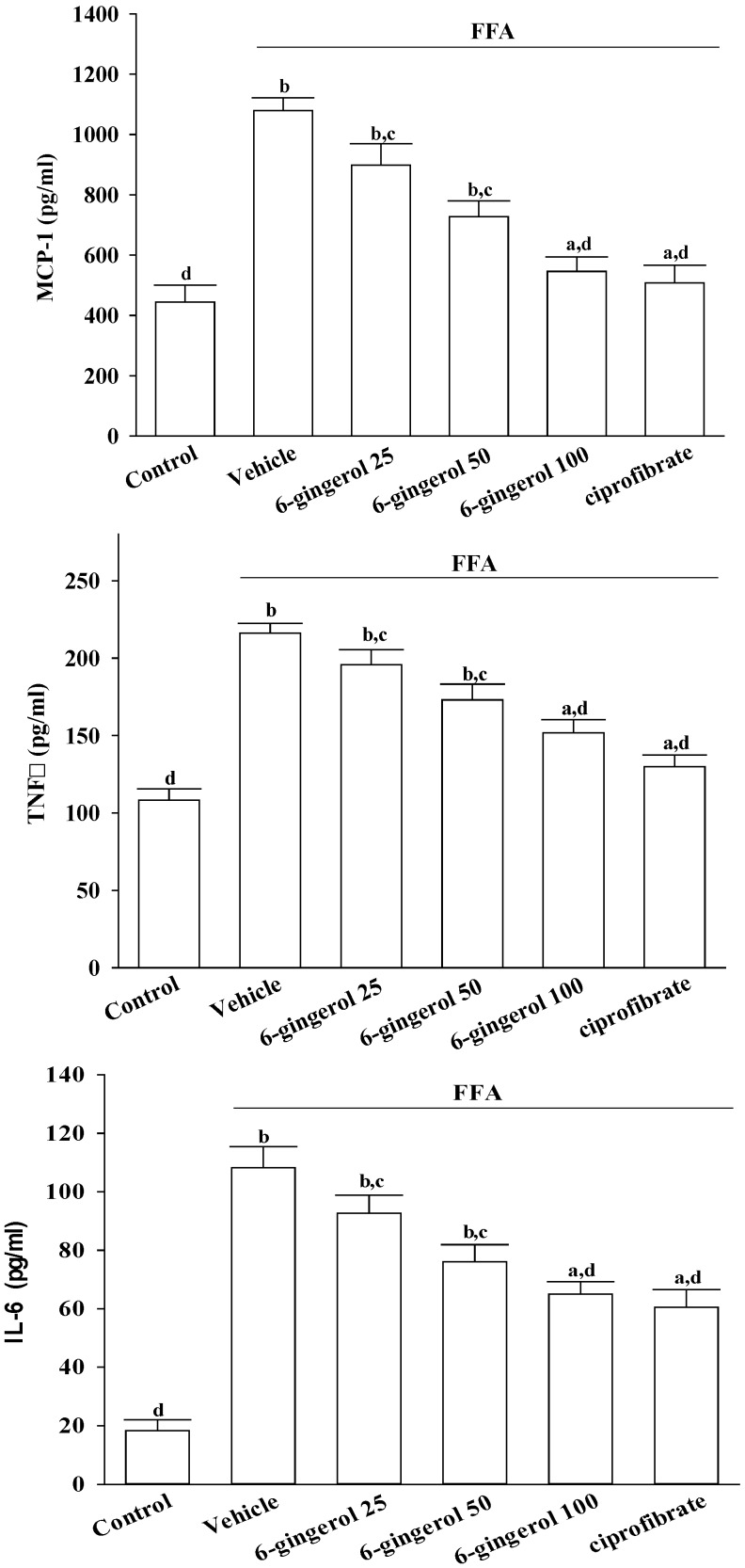
Different treatment effects on FFAs mixture-induced inflammatory cytokines overproduction in HepG2 cells. HepG2 cells were exposed to a FFAs mixture (0.33 mmol/L palmitate and 0.66 mmol/L oleate) for 24 h only or pre-incubated with 6-gingerol at concentrations of 25 μmol/L (6-gingerol 25), 50 μmol/L (6-gingerol 50), or 100 μmol/L (6-gingerol 100), or with 100 μmol/L of ciprofibrate (ciprofibrate). The vehicle (distilled water) used to prepare the test medication solutions was given at the same volume. MCP-1, TNF-α, and IL-6 concentrations in cell-free culture supernatants were determined by ELISA. Cells supplemented with medium without any treatment served as the control. The results are the means ± SD of eight experiments. ^a^ *p* < 0.05 and ^b^ *p* < 0.01 compared to the untreated control results. ^c^ *p* < 0.05 and ^d^ *p* < 0.01 compared to the results for FFAs mixture-treated cells.

Figure 3 shows that exposing HepG2 cells to a FFAs mixture significantly increased their production of MCP-1, TNF-α, and IL-6 relative to non-FFAs mixture treated cells. Pre-treatment with 6-gingerol (100 μmol/L) for 24 h significantly attenuated the FFAs mixture-induced production of all these inflammatory cytokines. Ciprofibrate (100 μmol/L) almost entirely eliminated the FFAs mixture-induced production of MCP-1, TNF-α, and IL-6 in HepG2 cells.

### 3.2. Effects on Body Weight and Liver Weight

Treatment with the MCD diet significantly decreased body weight and liver weight in mice compared with the MCS diet treatment (Table 1). Treating MCD diet-fed mice with 6-gingerol (100 mg/kg/day) did not result in any significant effects on body weight or absolute liver weight, while the change in the relative liver weight was significantly attenuated by treatment with 6-gingerol at 100 mg/kg/day (Table 1). Similar results were found for MCD diet-fed mice that were treated with ciprofibrate (10 mg/kg/day; Table 1).

**Table 1:** Effects of treatments on blood biochemistry and hepatic parameters in MCS-diet-or MCD-diet-fed mice receiving four-weeks of treatment.

Parameter	MCS Diet	MCD Diet				
	Vehicle	Vehicle	6-Gingerol (mg/kg/day)			Ciprofibrate
			25	50	100	(10 mg/kg/day)
Initial body weight (BW) (g)	21.13 ± 1.54	21.09 ± 1.28	21.17 ± 1.49	21.03 ± 1.37	21.14 ± 1.62	21.20 ± 1.81
Final BW (g)	28.47 ± 1.83	14.87 ± 1.42	15.17 ± 1.68	15.47 ± 1.53	15.21 ± 1.73	15.39 ± 1.84
Liver absolute weight (g)	1.41 ± 0.12	0.49 ± 0.06	0.52 ± 0.08	0.56 ± 0.06	0.58 ± 0.04	0.59 ± 0.06
Liver relative weight (%)	4.91 ± 0.27 ^c^	3.30 ± 0.31 ^a^	3.42 ± 0.26 ^a^	3.62 ± 0.22 ^a^	3.85 ± 0.25 ^a^	3.90 ± 0.29 ^a^
Plasma glucose (mg/dL)	95.12 ± 2.64 ^c^	81.17 ± 4.17 ^a^	82.24 ± 3.83 ^a^	84.45 ± 3.26 ^a^	83.33 ± 3.94 ^a^	81.26 ± 4.03 ^a^
Plasma TC (mg/dL)	153.90 ± 3.96 ^d^	25.39 ± 4.28 ^b^	24.36 ± 4.63 ^b^	24.26 ± 4.48 ^b^	25.65 ± 4.12 ^b^	26.52 ± 3.91 ^b^
Plasma TG (mg/dL)	98.81 ± 3.02 ^d^	52.36 ± 3.11 ^b^	57.52 ± 3.73 ^b^	53.91 ± 3.96 ^b^	52.49 ± 4.02 ^b^	56.30 ± 3.24 ^b^
Plasma ALT (U/L)	50.61 ± 6.27 ^d^	297.14 ± 16.23 ^b^	248.41 ± 15.78 ^b,c^	180.44 ± 14.56 ^b,c^	124.45 ± 12.47 ^a,d^	94.23 ± 10.87 ^a,d^
Plasma AST (U/L)	115.21 ± 10.91 ^d^	464.85 ± 17.79 ^b^	386.03 ± 16.55 ^b,c^	280.11 ± 17.35 ^b,c^	207.29 ± 18.40 ^a,d^	166.25 ± 13.24 ^a,d^
Hepatic TC (µmol/g liver)	12.23 ± 0.62 ^d^	20.64 ± 1.16 ^b^	18.82 ± 1.27 ^b,c^	17.04 ± 1.04 ^b,c^	15.22 ± 1.17 ^a,c^	14.56 ± 1.46 ^a,d^
Hepatic TG (µmol/g liver)	9.16 ± 0.82 ^d^	18.61 ± 1.28 ^b^	15.52 ± 1.17 ^b^	13.18 ± 1.51 ^b,c^	11.77 ± 1.09 ^a,c^	11.06 ± 1.21 ^d^

The vehicle (distilled water) used to prepare the tested medication solution was given at the same volume. Values (mean ± SD) were obtained from each group of eight animals in each group after four weeks of the experimental period. ^a^ *p* < 0.05 and ^b^ *p* < 0.01 compared to the values of vehicle-treated MCS-diet fed mice in each group, respectively. ^c^ *p* < 0.05 and ^d^ *p* < 0.01 compared to the values of vehicle-treated MCD-diet fed mice in each group, respectively.

### 3.3. Effects on Plasma and Hepatic Lipids in Mice

Glucose, TC, and TG plasma levels were lower in MCD diet-fed mice than those in MCS diet-fed animals. Treating MCD diet-fed mice with 6-gingerol (100 mg/kg/day) or ciprofibrate (10 mg/kg/day) had no significant effects on plasma glucose, TC, or TG levels (Table 1).

Hepatic TC and TG levels were significantly higher in MCD diet-fed mice as compared with MCS diet-fed mice, and these were reduced by 26.3% and 36.8%, respectively, in MCD diet-fed mice that were treated with 6-gingerol (100 mg/kg/day; Table 1). Ciprofibrate (10 mg/kg/day) treatment also reduced hepatic TC and TG levels to 29.5% and 40.6%, respectively, of those in vehicle-treated MCD diet-fed mice (Table 1).

### 3.4. Effects on Mouse Liver Injury

Plasma ALT and AST activities in MCD diet-fed mice were higher than those in MCS diet-fed mice (Table 1). ALT and AST activities were markedly reduced in MCD diet-fed mice that were treated for four weeks with a high-dose (100 mg/kg/day) of 6-gingerol (Table 1). Ciprofibrate (10 mg/kg/day) treatment also significantly attenuated the changes in plasma ALT and AST activities in MCD diet-fed mice (Table 1).

**Figure 4:**
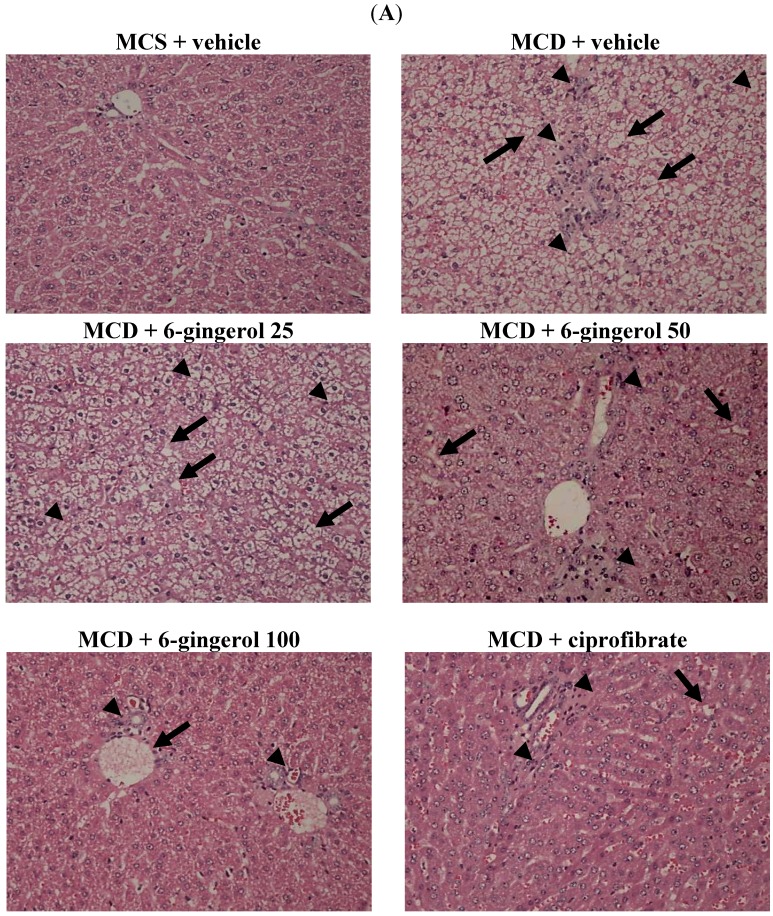

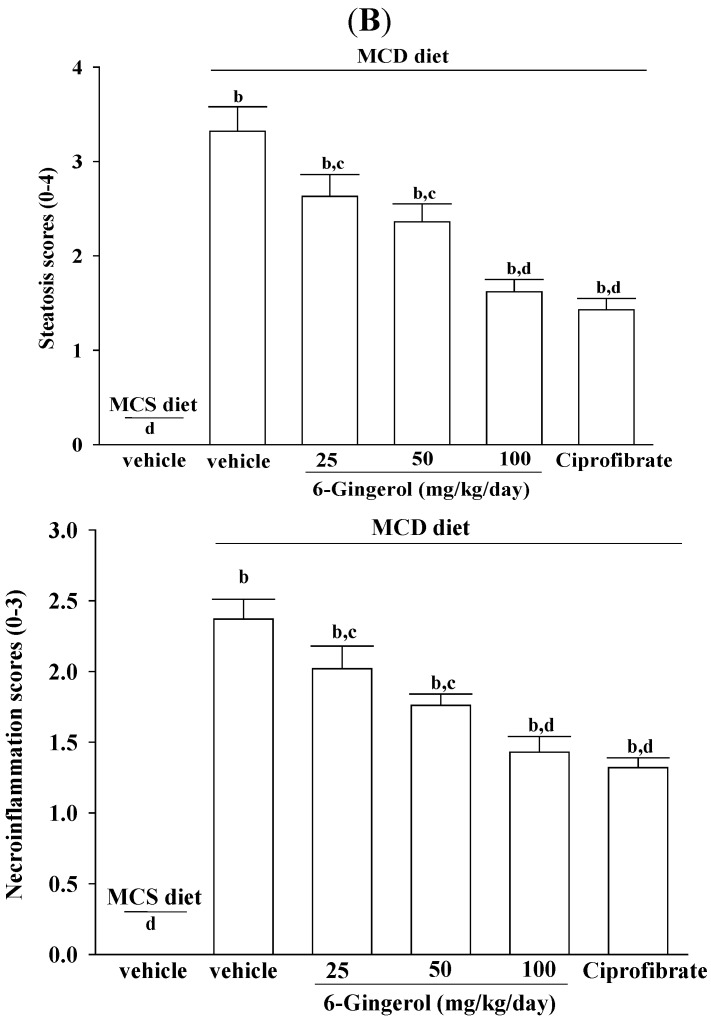
Effects of different treatments on liver histology. (**A**) Representative images of hematoxylin and eosin stained livers from MCS diet- and MCD diet-fed mice after four-weeks of 6-gingerol or ciprofibrate treatment. The vehicle (distilled water) used to prepare the test medication solutions was given at the same volume. Photomicrographs (original magnification, 200×) of tissues isolated from vehicle-treated MCS diet-fed mice (MCS + vehicle), vehicle-treated MCD diet-fed mice (MCD + vehicle), 25 mg/kg/day (MCD + 6-gingerol 25), 50 mg/kg/day (MCD + 6-gingerol 50), and 100 mg/kg/day (MCD + 6-gingerol 100) 6-gingerol-treated MCD diet-fed mice. Another group of MCD diet-fed mice was orally administered 10 mg/kg/day of ciprofibrate (MCD + ciprofibrate). Arrows and arrowheads indicate fat droplets and necro-inflammatory foci, respectively. (**B**) Hepatic steatosis and necro-inflammation scores. Results are means ± SD for eight mice/group after four weeks of treatment. ^a^ *p* < 0.05 and ^b^ *p* < 0.01 compared to the values of vehicle-treated MCS-diet fed mice. ^c^ *p* < 0.05 and ^d^ *p* < 0.01 compared to the values of vehicle-treated MCD-diet fed mice.

Feeding the MCD diet to mice resulted in a classical pathophysiological picture of NASH, with microvesicular and macrovesicular steatosis and multiple foci of inflammatory cell accumulation in the liver; these alterations were attenuated by treatment with 6-gingerol (Figure 4A). Ciprofibrate (10 mg/kg/day) treatment also improved hepatic steatosis and reduced hepatic necro-inflammation in MCD diet-fed mice (Figure 4A). Histological grading of liver sections confirmed that 6-gingerol and ciprofibrate attenuated both hepatic steatosis and necro-inflammation (Figure 4B).

### 3.5. Effects on Inflammatory Cytokines in Mice

**Figure 5:**
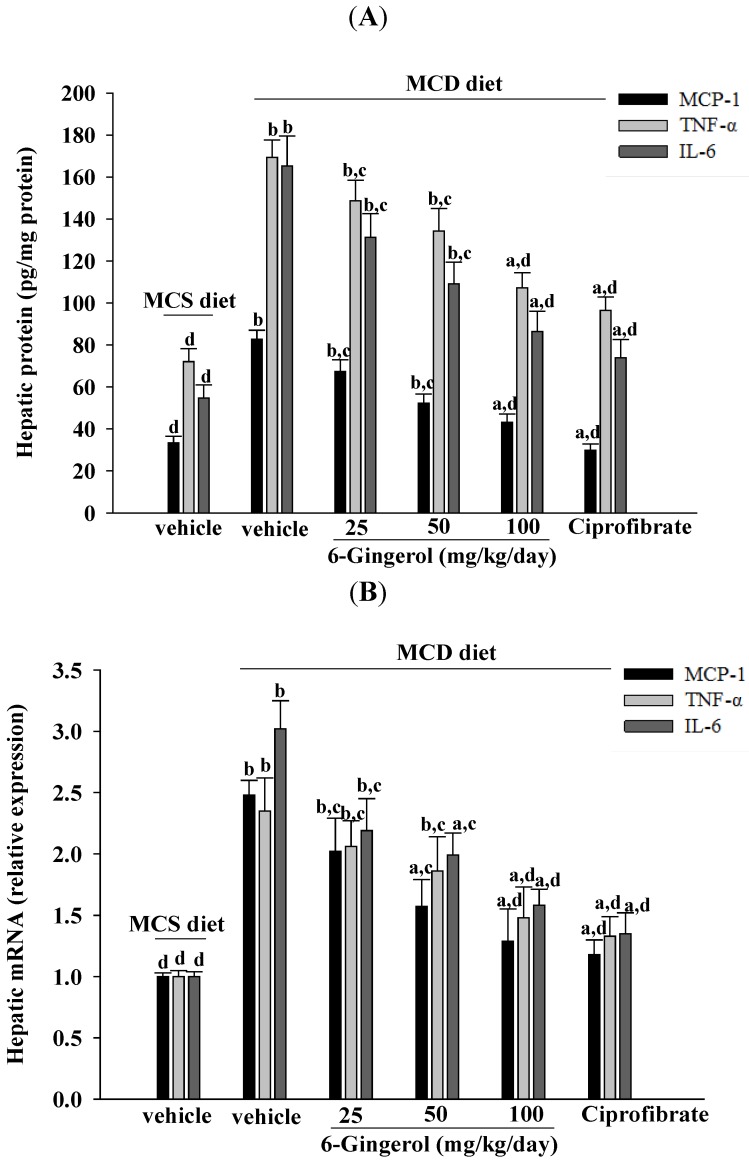
Effects of different treatments on inflammatory cytokines in mice. (**A**) Protein and (**B**) mRNA levels of MCP-1, TNFα and IL-6 in the livers of MCS diet- and MCD diet-fed mice after four weeks of treatment. MCD diet-fed mice were administered 25, 50, or 100 mg/kg/day of 6-gingerol or 10 mg/kg/day of ciprofibrate (ciprofibrate) by oral gavage once daily for four weeks. Another group of animals were administered the same volume of vehicle (distilled water) used to prepare the test medication solutions. The results are means ± SD for eight mice/group after four weeks of treatment. ^a^ *p* < 0.05 and ^b^ *p* < 0.01 compared to the values of vehicle-treated MCS-diet fed mice. ^c^ *p* < 0.05 and ^d^ *p* < 0.01 compared to the values of vehicle-treated MCD-diet fed mice.

MCD diet-fed mice had higher hepatic protein and mRNA MCP-1, TNF-α, and IL-6 levels as compared to those of MCS diet-fed mice (Figure 5). Both the protein and mRNA levels of hepatic cytokines in 6-gingerol (100 mg/kg/day)-treated MCD diet-fed mice were lower than those of their vehicle-treated counterparts (Figure 5). Ciprofibrate (10 mg/kg/day) treatment also reduced hepatic cytokine protein and mRNA levels as compared to those of MCS diet-fed mice (Figure 5).

### 3.6. Effects on Hepatic IκBα and NF-κB Expression and NF-κB Binding Activity

**Figure 6:**
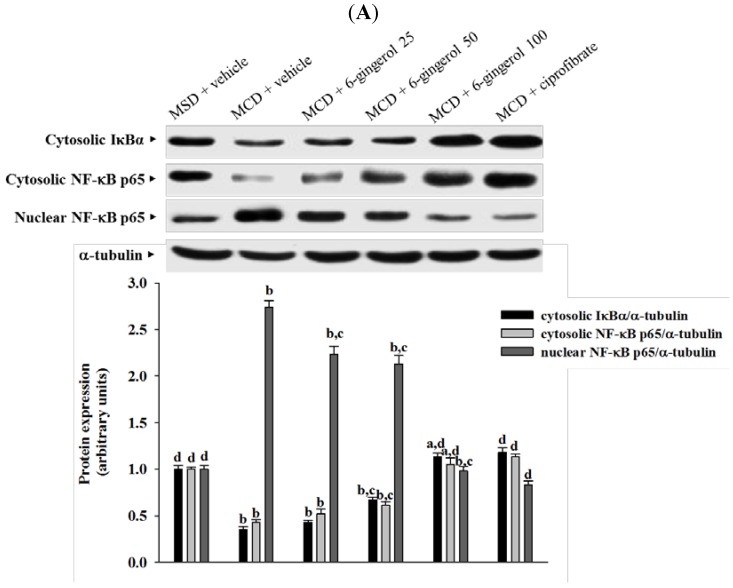

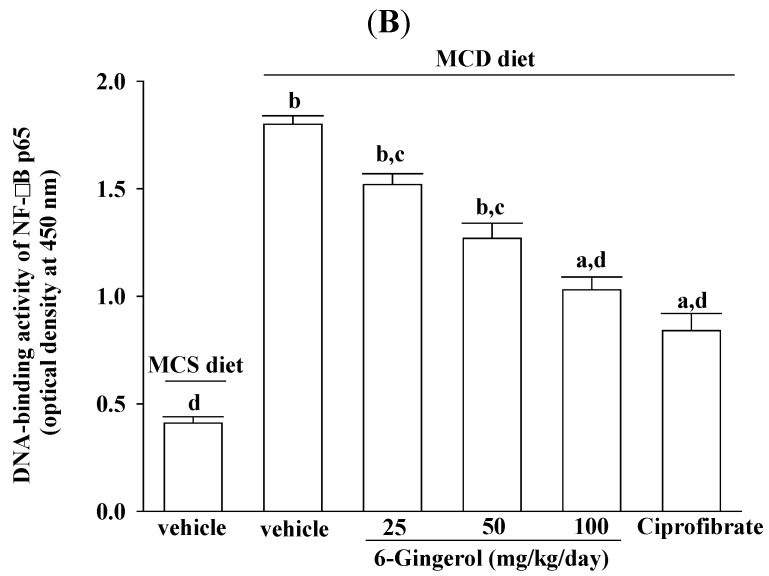
Different treatment effects on IκBα and NF-κB expression and NF-κB binding activity in liver tissue. (**A**) Protein expression of IκBα and NF-κB p65, and (**B**) DNA binding activity of NF-κB p65 in the livers of MCS diet- and MCD diet-fed mice after four weeks of treatment. MCD diet-fed mice were administered either 6-gingerol at 25 mg/kg/day (MCD + 6-gingerol 25), 50 mg/kg/day (MCD + 6-gingerol 50), or 100 mg/kg/day (MCD + 6-gingerol 100) or 10 mg/kg/day of ciprofibrate (MCD + ciprofibrate) by oral gavage once daily for four weeks. Additional groups of MCD diet-fed mice (MCD + vehicle) and MSD-fed mice (MSD + vehicle) were administered the same volume of vehicle (distilled water) used to prepare the test medication solutions. The results are means ± SD for eight mice/group after four weeks of treatment. ^a^ *p* < 0.05 and ^b^ *p* < 0.01 compared to the values of vehicle-treated MCS-diet fed mice. ^c^ *p* < 0.05 and ^d^ *p* < 0.01 compared to the values of vehicle-treated MCD-diet fed mice.

Cytosolic IκBα protein expression in the livers of MCD diet-fed mice was reduced to 35.7% that in MCS diet-fed mice, and 6-gingerol treatment reduced IκBα degradation in a dose-dependent manner (Figure 6A). Treating MCD diet-fed mice with ciprofibrate (10 mg/kg/day) increased the cytosolic IκBα protein level to near that in MCS diet-fed mice (Figure 6A).

The hepatic level of cytosolic NF-κB p65 protein in MCD diet-fed mice was clearly lower than that in MCS diet-fed mice, and was up-regulated after treatment with 6-gingerol (100 mg/kg/day) or ciprofibrate (10 mg/kg/day), which resulted in increases of 244.1% and 262.7%, respectively (Figure 6A). MCD diet-induced upregulation of nuclear NF-κB p65 protein was reduced by 35.7% and 30.3% relative to that in vehicle-treated MCD diet-fed mice after four weeks of treatment with 6-gingerol (100 mg/kg/day) or ciprofibrate (10 mg/kg/day), respectively (Figure 6A).

The DNA binding activity of NF-κB p65 was significantly greater in the livers of MCD diet-fed mice compared to that in MCS diet-fed mice (Figure 6B). 6-Gingerol (100 mg/kg/day)-treated MCD diet-fed mice had 45.3% lower NF-κB p65 binding activity in the liver compared to that of their vehicle-treated counterparts (Figure 6B). Ciprofibrate (10 mg/kg/day) suppressed hepatic NF-κB p65 binding activity of MCD diet-fed mice by 56.5% relative to that of their untreated counterparts (Figure 6B).

### 3.7. Effects on Hepatic mRNA Expression of Lipid Metabolism-Associated Genes

**Figure 7:**
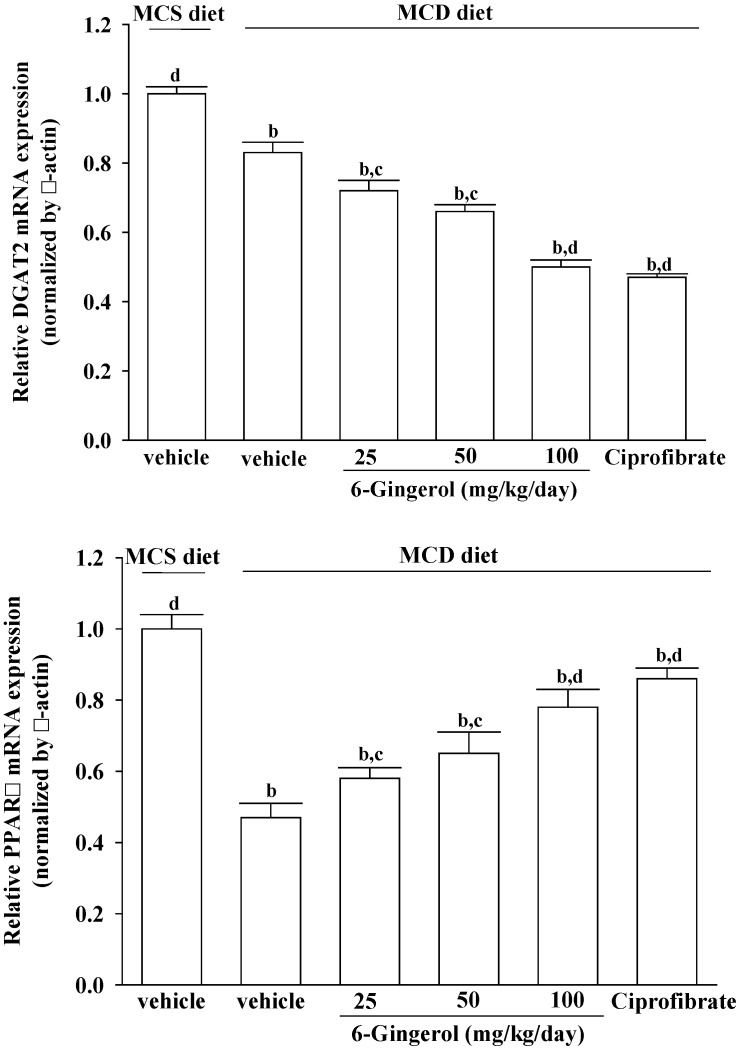
Effects of different treatments on DGAT2 and PPARα mRNA expression in liver tissue. MCD diet-fed mice were administered either 25, 50, or 100 mg/kg/day of 6-gingerol or 10 mg/kg/day of ciprofibrate (ciprofibrate) by oral gavage once daily for four weeks. Another group of mice were administered the same volume of vehicle (distilled water) used to prepare the test medication solutions. The results are means ± SD for eight mice/group after four weeks of treatment. ^a^ *p* < 0.05 and ^b^ *p* < 0.01 compared to the values of vehicle-treated MCS-diet fed mice. ^c^ *p* < 0.05 and ^d^ *p* < 0.01 compared to the values of vehicle-treated MCD-diet fed mice.

Administering 6-gingerol (100 mg/kg/day) to MCD diet-fed mice for four weeks resulted in a 43.3% down-regulation of hepatic DGAT2 mRNA levels relative to that in vehicle-treated counterparts (Figure 7). Hepatic PPARα mRNA levels in MCD diet-fed mice were clearly lower than those in MCS diet-fed mice, and were up-regulated by 6-gingerol treatment (165.7% increase; Figure 7). Similar results were obtained for ciprofibrate (10 mg/kg/day)-treated MCS diet-fed mice (Figure 7).

## 4. Discussion

Changes in the FFAs contents in the liver may affect lipid metabolism and inflammation [19,20]. Elevated FFAs are thus considered to be a major cause of liver cell injury and death in NASH [21]. Palmitic and oleic acids are common food constituents and are the most abundant FFAs among liver TGs in both healthy subjects and patients with NAFLD. In this study, a hepatocellular steatotic model was established using a mixture of oleate and palmitate [11]. Our results demonstrated that pre-incubating HepG2 cells with 6-gingerol significantly attenuated their overproduction of cellular fatty droplets and inflammatory cytokines after their exposure to an excess FFAs mixture. These results indicated that 6-gingerol might be effective for preventing and/or reversing lipid accumulation and inflammatory responses, which may accelerate liver injuries in NAFLD/NASH.

Using a diet that is deficient in essential amino acids, such as methionine and choline, is a well-accepted model for inducing NASH, which recapitulates many of the features of this disease in humans, including a histological picture that mimics that seen in human fibrotic disorders associated with hepatic lipid accumulation and the occurrence of inflammation and oxidative stress [13]. To support the use of 6-gingerol for treating NASH-like disorders, we investigated the hepatotherapeutic effects of 6-gingerol in an animal model of MCD diet-induced hepatic injury. Mice consistently developed steatosis and inflammatory cell infiltration after ingesting the MCD diet, in line with a previous report on this nutritional model of NASH [22]. The accumulation of fat in the liver resulting from choline deficiency occurs because choline is required to make the phosphatidyl-choline portion in very low-density lipoprotein (VLDL) particles [23,24]. In the absence of choline, VLDL is not secreted and TG builds up in the liver cytosol. These earlier findings are in agreement with our data, where the plasma levels of TC and TG are low, but lipid accumulation in the liver is extensive. We found that 6-gingerol exerted pharmaceutical effects on hepatic TC and TG levels. In addition, the liver histopathological findings of MCD diet fed-mice were considerably ameliorated by 6-gingerol treatment. These results indicate that 6-gingerol may provide a novel therapeutic strategy for NASH.

With regard to changes in the lipid metabolism that are caused by fat accumulation, liver aminotransferase activity is altered. Assessing the damage caused by fat accumulation in the liver is important for the diagnosis of NAFLD/NASH [25]. ALT and AST are considered to be sensitive indicators of hepatocellular damage and, within certain limits, can provide a quantitative evaluation of the degree of liver damage [25]. Although the plasma levels of ALT and AST are still higher than those of MCS diet-fed mice, ALT and AST activities were markedly reduced in MCD diet-fed mice that were treated with 6-gingerol. The hepatotherapeutic effect of 6-gingerol in an MCD diet-induced NASH animal model was thus considerable.

Inflammation is thought to be the driving force behind NASH, the progression to fibrosis, and subsequent cirrhosis [26]. The results of the current study demonstrated that 6-gingerol treatment reduced hepatic MCP-1, TNF-α, and IL-6 protein levels that had been elevated after ingesting the MCD diet. These results support the proposition that the therapeutic effect of 6-gingerol on NASH may be associated with regulating inflammatory cytokines. NF-κB, a redox-sensitive transcription factor that regulates numerous inflammatory genes, has been implicated in the development of numerous pathological states. NFκB activation induces gene expression, which results in the transcription of factors that promote inflammation, such as leukocyte adhesion molecules, cytokines, and chemokines [27]. Cytosolic NF-kB activation is tightly regulated by its inhibitory protein IκB, and involves the phosphorylation, ubiquitination, and proteolysis of IκB [28]. IκB degradation results in the nuclear translocation of NF-κB, where it binds to gene promoter sites for their transcription [28]. In the present study, 6-gingerol increased cytoplasmic IκB protein levels and significantly inhibited NF-κB nuclear translocation in the livers of MCD diet-fed mice. 6-Gingerol also suppressed the upregulation of nuclear NF-κB DNA binding activity in the livers of MCD diet-fed mice, suggesting that 6-gingerol acted against the NF-κB activation induced by the MCD diet. Consistent with the reduction in NF-κB activation, the mRNA expressions of MCP-1, TNF-α, and IL-6 were lower in MCD diet-fed mice that were treated with 6-gingerol, which indicated that the protective effects of 6-gingerol occurred at the transcriptional level. 6-Gingerol protects the HuH7 human hepatoma cells against IL-1β-induced inflammatory insults through inhibition of the reactive-oxygen-species-activated NF-κB/cyclooxygenase-2 pathway [9]. Our results support previous findings and demonstrate that the anti-inflammatory effect of 6-gingerol on MCD diet-induced NASH is mediated, in part, through the NF-κB signaling pathway.

MCD diet promotes intrahepatic lipid accumulation through impairment of VLDL secretion [23,24]. However, the mRNAs encoding genes related to lipogenesis, such as DGAT2, were suppressed in mice fed a MCD diet [29,30], which is in agreement with our findings. DGAT2 is a microsomal enzyme that promotes the binding of acyl-CoA to 1,2-diacylglycerol, and thus constitutes the final step in TG biosynthesis [31]. Our results show that DGAT2 expression in MCD diet-fed mice also decreased in the 6-gingerol-treated group. The role of 6-gingerol in the inhibition of hepatic lipid accumulation in MCD diet-fed mice thus needs to be further clarified.

PPARα plays a central role in the β-oxidation of fatty acids, particularly in the liver [32,33]. Activation of the PPARα receptor results in increased transcription of those genes related to fatty acid transport across the cell membrane, intracellular lipid trafficking, mitochondria and peroxisome fatty acid uptake, and mitochondria and peroxisome fatty acid β-oxidation [34]. PPARα reportedly protects against high-fat or MCD-induced NASH in rodents [35,36]. Ciprofibrate, a fibric acid derivative, is a commercially available drug used for treating hyperlipidemia in patients with and without coronary artery disease [37]. Although the effect of 6-gingerol was less pronounced than that of ciprofibrate, PPARα mRNA levels were significantly increased in 6-gingerol-treated and MCD diet-fed mice. These results suggest that 6-gingerol may be related to the suppressed β-oxidation of fatty acids in NASH model mice. 6-Gingerol may thus be a suitable therapeutic adjunct for patients who are particularly sensitive to fibrate-associated side effects [38]. Due to different lipid metabolisms in humans and mice, the results from mice studies cannot be generalized to humans. Placebo controlled human studies are thus required to find the usability of 6-gingerol in human NASH indications.

## 5. Conclusions

Our results provide novel evidence that 6-gingerol protected against MCD-induced hepatic inflammation by decreasing the induction of inflammatory cytokine genes via an NF-κB-dependent pathway. Additionally, 6-gingerol had a repressive effect on hepatic steatosis, which was associated with inducing PPARα expression. These results suggest that 6-gingerol is a food component that may protect against nutritional steatohepatitis.
